# Anchor Clustering for million-scale immune repertoire sequencing data

**DOI:** 10.1186/s12859-024-05659-z

**Published:** 2024-01-25

**Authors:** Haiyang Chang, Daniel A. Ashlock, Steffen P. Graether, Stefan M. Keller

**Affiliations:** 1 Department of Mathematics and Statistics, University of Guelph, 50 Stone Rd E, Guelph, ON N1G 2W1 Canada; 2 Department of Molecular and Cellular Biology, University of Guelph, 50 Stone Rd E, Guelph, ON N1G 2W1 Canada; 3 Department of Pathology, Microbiology and Immunology, School of Veterinary Medicine, University of California Davis, One Shields Avenue, Davis, CA 95616 USA

**Keywords:** Unsupervised clustering, Immune repertoire, Clonal relationship, Lymphocyte antigen receptor

## Abstract

**Background:**

The clustering of immune repertoire data is challenging due to the computational cost associated with a very large number of pairwise sequence comparisons. To overcome this limitation, we developed Anchor Clustering, an unsupervised clustering method designed to identify similar sequences from millions of antigen receptor gene sequences. First, a Point Packing algorithm is used to identify a set of maximally spaced anchor sequences. Then, the genetic distance of the remaining sequences to all anchor sequences is calculated and transformed into distance vectors. Finally, distance vectors are clustered using unsupervised clustering. This process is repeated iteratively until the resulting clusters are small enough so that pairwise distance comparisons can be performed.

**Results:**

Our results demonstrate that Anchor Clustering is faster than existing pairwise comparison clustering methods while providing similar clustering quality. With its flexible, memory-saving strategy, Anchor Clustering is capable of clustering millions of antigen receptor gene sequences in just a few minutes.

**Conclusions:**

This method enables the meta-analysis of immune-repertoire data from different studies and could contribute to a more comprehensive understanding of the immune repertoire data space.

**Supplementary Information:**

The online version contains supplementary material available at 10.1186/s12859-024-05659-z.

## Background

Adaptive immunity has evolved to recognize and combat a vast array of pathogens. A key mechanism of this flexible immune response is to generate a broad repertoire of highly polymorphic antigen receptors in lymphocytes by rearrangement of gene segments and the addition of random nucleotides. B cell receptors (BCRs), also known as immunoglobulins or antibodies, mediate humoral immunity by recognizing soluble antigens [1]. The possible number of unique human BCR sequences is estimated at 10^12^ [2] for the naive repertoire. This diversity is further amplified when activated B cells undergo somatic hypermutation during affinity maturation, theoretically elevating the diversity beyond 10^14^ [3].

The complementarity determining region 3 (CDR3) is the most polymorphic part of the B and T cell receptor and determines antigen specificity; the resulting lymphocytes can recognize a wide range of distinct epitopes, and hence a diversified immune repertoire [4]. A challenge in understanding immune repertoires is the fact that different antigen receptor sequences can bind to the same epitope [5–7], resulting in a many-to-one relationship between the antigen receptor sequence and the cognate antigen. Antigen receptor sequences that exhibit similar junctional regions in the same individual are considered to have descended from a shared lymphocyte ancestor, indicating clonal relatedness [8, 9]. In addition, identical or similar clonotypes can occur in different individuals, a phenomenon termed "public clonotypes". To characterize clonal relatedness and identify public clonotypes, it is desirable to find an efficient method to cluster similar antigen receptor sequences based on the presumed epitope specificity.

In recent years, several methods have been described for the clustering of immune repertoire data. In general, clustering methods differ based on whether they process nucleotide [10–13] or amino acid sequences [14–18], and whether they target BCR or T cell receptor (TCR) sequences. Typically, methods for TCR clustering use amino acid sequence data, while methods for BCR data use nucleotide sequences. The reason is that the tracing of clonal evolution during affinity maturation is relevant for BCR data only and requires a nucleotide-level resolution to detect synonymous substitutions. Moreover, with the appearance of the therapeutic structural antibody database, clustering BCR based on their amino acid data would be meaningful in investigating the structural diversity of repertoires and developing applications in immunodiagnostics and immunotherapeutics [19, 20].

To identify related antigen receptor sequences, distance-dependent similarity metrics such as Hamming distance (HD) [12, 13] and Levenshtein distance [11, 21, 22] are commonly applied by pairwise sequence comparison of junctional sequences. Because the time for calculating all-versus-all comparisons rises quadratically with the number of antigen sequences (*O(n2)*), determining the genetic distance between all sequences using either method is not feasible for millions of nucleotide sequences. Consequently, a clustering algorithm that circumvents the need for an all-versus-all sequence comparison would significantly improve clustering performance.

Here we present Anchor Clustering, a novel approach for clustering of BCR nucleotide sequence data. Anchor Clustering incrementally partitions a dataset based on genetic distance to maximally spaced anchor sequences until each cluster is small enough to be amenable to pairwise sequence comparison. We show that Anchor Clustering can cluster datasets up to 10 times faster than existing methods at comparable clustering quality. In addition, Anchor clustering can cluster datasets with millions of sequences with minimal hardware requirements. This enables the meta-analysis of immune-repertoire data from different studies and could contribute to a more comprehensive understanding of the immune repertoire data space.

## Methods

### Data

#### Simulated BCR data

Simulated immune repertoire data with known clonal relationships (previously described in [12, 23]) were used in this work. These datasets were generated based on the identified lineage tree topologies of four individuals (M2, M3, M4 and M5) with multiple sclerosis (MS) by picking a new germline sequence at random for each lineage and stochastically reintroducing mutations throughout the lineage branches [24]. This process was repeated 10 times for each individual, resulting in a total of 40 simulations (dataset group ‘*MS*’). In addition, we created 15 composite datasets by merging all 40 simulated MS datasets, discarding ambiguous and duplicate sequences, and extracting 10 kilo (K), 50 K and 100 K sequences with junctional lengths of 48, 51, 54, 57, and 60 nucleotides, respectively (dataset group ‘*MS-Mixed*’). The details of each dataset and its usage are further summarized in Additional file 1.

#### Experimental BCR data

Experimental BCR repertoire data were obtained from the iReceptor platform [25] and are summarized in Additional file 1. To benchmark clustering speed, antigen receptor gene sequences from COVID-19 positive individuals and healthy controls [26] were randomly selected and combined into 12 datasets ranging from 10 to 300 K sequences with ambiguous and duplicate sequences removed (dataset group ‘*C19*’). To test clustering performance on million-scale datasets, sequences from four different datasets (hepatitis B [27], chronic lymphocytic leukemia [28], systemic lupus erythematosus, and COVID-19 [26, 29]) were randomly selected and combined at equal proportions to generate datasets ranging from 1 million (M) to 4 M unique sequences (dataset group ‘*4 Diseases Mixed*’).

### Anchor Clustering workflow

#### Overview

**Fig. 1:**
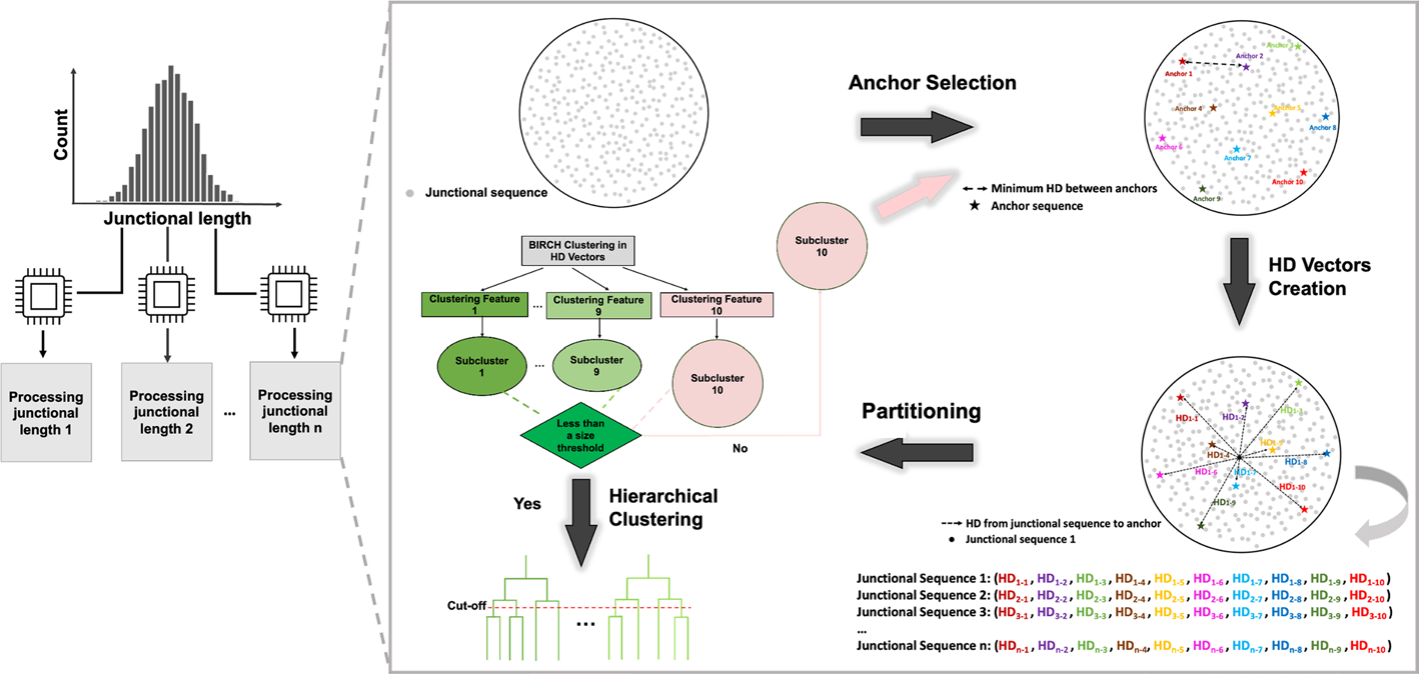
Anchor Clustering workflow. Sequences are grouped by junctional length and processed independently. Anchor sequences are selected using a Point Packing algorithm, which ensures that the Hamming distance (HD) between any two selected anchors satisfies a minimum distance requirement. For every sequence, a distance vector is created by evaluating the HD of the sequence with every anchor. Next, the dataset is partitioned into subclusters based on the HD vectors using the BIRCH algorithm. If any of the resulting clusters is below a size threshold, sequences are clustered using single linkage hierarchical clustering. If a subcluster is larger than the size threshold, the process is repeated until all subclusters meet the size requirement

Anchor Clustering integrates a Point Packing algorithm for anchor selection and unsupervised learning for partitioning of sequences for rapid and accurate clustering (Fig. 1**)**. After stratifying junctional sequences by length, several maximally spaced sequences are picked as anchor sequences using a Point Packing algorithm. Then, the HD to all anchor sequences is calculated for each remaining sequence and distances are concatenated into a distance vector. The dataset is then subdivided based on the distance vectors using the BIRCH algorithm. This workflow is repeated iteratively for each generated subcluster until every subcluster is smaller than a user-defined size threshold. Lastly, single linkage hierarchical clustering is performed on each remaining subcluster, and the resulting clusters are cut using a user-defined threshold.

#### Anchor selection

Anchor sequences were selected using a Point Packing algorithm as described previously [30–32]. In short, Conway’s Lexicode Algorithm (CLA) takes a subset of points from a metric space, along with a specified minimum distance constraint, and produces the output set that satisfies the minimum distance requirement. In this study, junctional sequences represented points and the HD was used as a distance measure. Conway’s Variation Operator (CVO) takes two subsets of packings with minimum distance constraints as the input and returns one set of packing as the output. Specifically, the union of these two subsets is augmented with random points, which are shuffled and run through CLA to produce a new collection of points, still obeying the minimum distance constraint. Details of the CLA and CVO algorithms can be found in Additional file 2.

The Point Packing algorithm requires three user-defined hyperparameters—population size, random material rate and minimum distance. Population size refers to the initial number of anchor sets. The random material rate is analogous to mutation and controls the number of sequences to be introduced. Minimum distance refers to the minimum HD between anchors and is dependent on the junctional length of sequences. To normalize the minimum distance parameter with respect to junctional length, we introduced a minimum distance ratio defined as minimum distance/junctional length. The following parameters were tested: minimum distance ratios {0.5, 0.6, 0.7, 0.8 and 0.9}; population sizes {100, 250, 500, 750 and 1000}; random material rates {10, 25, 50, 75 and 100}. This resulted in a total of 125 parameter combinations (5 × 5 × 5), which were applied to all 15 datasets of the dataset group ‘*MS-Mixed’*.

#### Partitioning into subclusters

After identifying anchor sequences, the distance of each remaining sequence to all anchor sequences was determined and the distance results were concatenated into a distance vector for each sequence. The dataset was then partitioned based on distance vectors using the BIRCH algorithm [33]. The BIRCH algorithm iteratively divides a single cluster into smaller clusters and requires the specification of a cluster number parameter and a radius threshold. The number of clusters was set to the number of anchor sequences selected during Point Packing. The radius threshold determines whether a new sequence is merged into an existing subcluster or becomes a new subcluster. To characterize the effect of the radius parameter and anchor number on the clustering performance, the minimum distance ratios {0.5, 0.6, 0.7, 0.8 and 0.9} and radius parameters {0.01, 0.05, 0.1 and 0.5} were tested on four simulated MS datasets (*M2-1, M3-1, M4-1 and M5-1*).

#### Hierarchical clustering of subclusters

Anchor selection and data partitioning are repeated iteratively until the number of sequences within a given subcluster falls below a user-defined size threshold. Sequences are then clustered using single linkage-based hierarchical clustering with a user-define normalized HD cut-off. To evaluate the effect of the subcluster size threshold on clustering performance and speed, size thresholds {1000, 2000, 3000, 4000 and 5000} were applied to the four simulated MS datasets (*M2-1, M3-1, M4-1* *and* *M5-1*) utilizing several minimum distance ratios {0.5, 0.6, 0.7 and 0.8}. To determine an optimal normalized HD threshold, we assessed clustering performance for different normalized HD values ranging from 0.1 to 0.2 with increments of 0.01.

### Clustering performance evaluation

#### Simulated BCR data

The clustering quality of Anchor Clustering was evaluated based on sensitivity, precision, and F-measure using the 40 simulated MS datasets. True positives (TP) were defined as clonally related sequence pairs that were correctly clustered in the same cluster. False Negatives (FN) were defined as clonally related sequence pairs that were falsely separated into different clusters. False positives (FP) were defined as clonally unrelated sequence pairs that were falsely clustered in the same cluster. Sensitivity (recall) and precision (positive predictive value) were calculated as follows:

$$Sensitivity=\frac{TP}{TP +FN}$$

$$Precision=\frac{TP}{TP +FP}$$

The F measure (also known as the F score) was defined as the harmonic mean of sensitivity and precision.

$$FM=\frac{2*Sensitivity*Precision}{Sensitivity +Precision}$$

#### Experimental BCR data

Given that the clonal relationships in the experimental datasets are unknown, the evaluation of clustering quality was carried out using retention and fraction metrics. A cluster was considered ‘pure’ if all sequences in a given cluster had the same disease label. If a cluster contained a single sequence only (i.e., a singleton), it was considered a pure cluster. Singleton retention, i.e., the percentage of singletons in a dataset, was calculated by dividing the number of singletons by the total number of sequences.

$$Singleton\; Retention=\frac{Singletons}{Total\; sequences}$$

Similarly, the singleton fraction was calculated as the percentage of singletons divided by the total number of clusters, providing a measure of the proportion of clusters that were represented by singleton sequences.

$$Singleton\; Fraction=\frac{Singletons}{Total\; clusters}$$

Non-singleton retention was defined as the percentage of junctional sequences that appeared in all non-singleton pure clusters, divided by the total number of sequences.

$$Non-singleton\; Retention=\frac{Sequences\; in\; all\; non-singleton\; pure\; clusters}{Total\; sequences}$$

Non-singleton fraction was calculated as the ratio of the number of non-singleton pure clusters to the total number of generated clusters, expressed as a percentage.

$$Non-singleton\; Fraction=\frac{Pure\; non-singleton\; clusters}{Total\; clusters}$$

#### Benchmarking Anchor Clustering

To benchmark its performance, we compared Anchor Clustering to three existing BCR nucleotide sequence clustering tools: DefineClones [10], SCOPe [13] and an Alignment free method [34]. DefineClones uses a hierarchical-based clustering method with a specified linkage method and forms clusters using a bimodal distribution-determined threshold. SCOPe is a spectral-based clustering method with an adaptive threshold to determine the local sequence neighborhood. The Alignment free method is based on natural language processing methodology and uses k-mer representations and re-weighting based on a numeric statistic reflecting the importance of k-mers in a sequence. The following distance clustering thresholds were tested: DefineClones with normalized HD {0.06, 0.08, 0.1, 0.12, 0.14 and 0.16}; SCOPe with upper-limit distance {0.04, 0.06, 0.08, 0.1 and 0.12}; Alignment free with cosine similarity {0.16, 0.18, 0.2, 0.22, 0.24 and 0.26}. The distance clustering threshold that achieved maximum retention was chosen as the optimal threshold for each method and was used for comparing pure and non-pure clusters of classifying COVID-19 and Healthy labeled sequences in the dataset ‘*C19-K_Healthy_*100K’. For parameters other than distance clustering thresholds, the default settings were used.

Runtime comparisons were performed using all datasets of the ‘*C19*’ dataset group and clustering performance (fraction and retention) was evaluated using the ‘*C19- K_ Healthy_100K’* dataset. Benchmarking was performed on a MacBook Air computer equipped with an Apple M2 chip and 16 GB memory.

To further optimize the speed and quality of Anchor Clustering on million-scale datasets, we explored the benefit of grouping sequences based on variable (V) and joining (J) gene usage before clustering (VJ grouping pre-clustering, shortened to VJ-Pre) as opposed to the workflow of grouping based on VJ usage after clustering (VJ grouping post-clustering, shortened to VJ-Post). If a junctional sequence had multiple V or J annotations, a cluster was greedily expanded if junctional sequences had at least one shared gene segment. Two Anchor Clustering strategies with normalized HD clustering thresholds from 0.08 to 0.16 with 0.02 increments were applied for achieving maximum non-singleton retention on the ‘*C19-K_Healthy_100K’* dataset. To reduce memory requirements of clustering million-scale datasets (dataset group ‘*4 Diseases Mixed’*), we applied a 10% fraction of data levels to be trained in their BIRCH models.

## Results

### Parameter optimization

#### Point Packing parameter settings

First, we assessed the effect of junctional length on anchor number and runtime and found that the junctional length had a minor influence on both anchor number and runtime (Additional file 3: Fig. S1). This result was consistent across 15 composite datasets (dataset group ‘*MS-Mixed*’), regardless of dataset size. Given the negligible impact of junctional length on the results of Point Packing, subsequent results were represented as an average across all junctional lengths.

Next, we explored the effect of minimum distance ratio, population size and random material rate on the number of generated anchors and runtime (Additional file 3: Fig. S2). The minimum distance ratio had the most significant effect on both the number of generated anchors and runtime. The smaller the distance ratio, the higher the number of generated anchors and the longer the runtime. The random material rate had a minor effect on the number of generated anchors but increased the runtime for the minimum distance ratios between 0.7 and 0.9. Based on these findings, a population size of 1000 and a random material rate of 0.5 were utilized as the default Point Packing parameters, because these settings provided a reasonable balance between anchor number and runtime.

#### BIRCH parameter settings

**Fig. 2:**
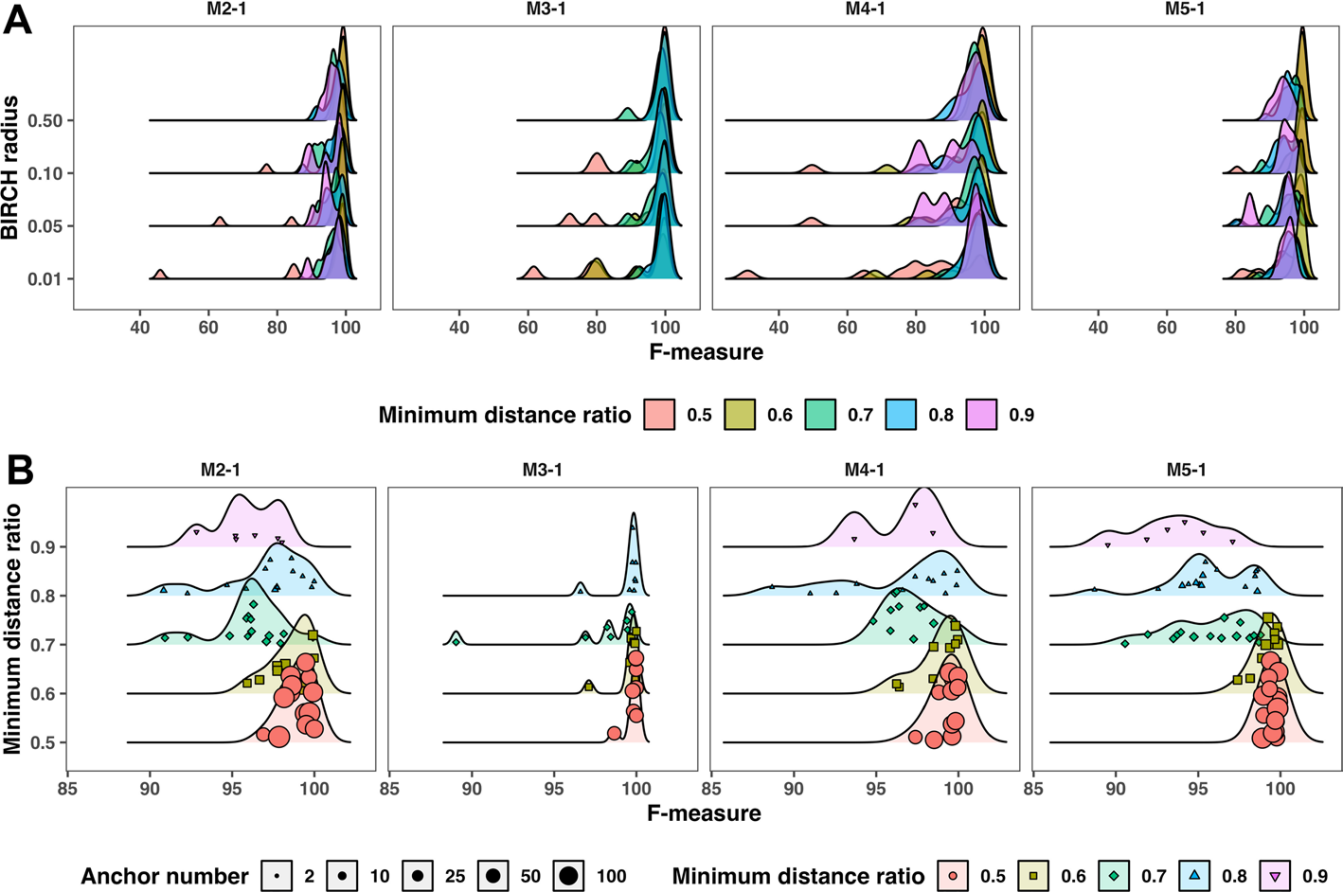
**A** Clustering performance (F-measure) of four simulated MS datasets (*M2-1, M3-1, M4-1* *and* *M5-1*) under four minimum distance ratios {0.5, 0.6, 0.7 and 0.8} and BIRCH radii {0.01, 0.05, 0.1 and 0.5}. **B** Clustering performance (F-measure) of four MS datasets (*M2-1, M3-1, M4-1* *and* *M5-1*) under four minimum distance ratios {0.5, 0.6, 0.7 and 0.8} with the same BIRCH radius 0.5

After generating anchor sequences by Point Packing, Anchor Clustering partitions the data into clusters using the BIRCH algorithm, which requires the definition of a cluster number parameter and a radius threshold. Since the cluster number is defined by the number of generated anchors, the radius threshold was the only parameter that required optimization. A radius threshold of 0.5 yielded superior clustering performance compared to lower radii (0.01, 0.05 and 0.1) (Fig. 2A). Of note, the poorer performance for lower radius values was associated with lower minimum distance ratios. When using a radius threshold of 0.5, a robust clustering performance with F-measure values consistently exceeding 85% was observed across all four datasets, irrespective of the minimum distance ratio (Fig. 2B). Minimum distance ratios of 0.5 and 0.6 resulted in higher anchor numbers and F-measure values greater than 95% across all four datasets.

#### Cluster size threshold settings

**Fig. 3:**
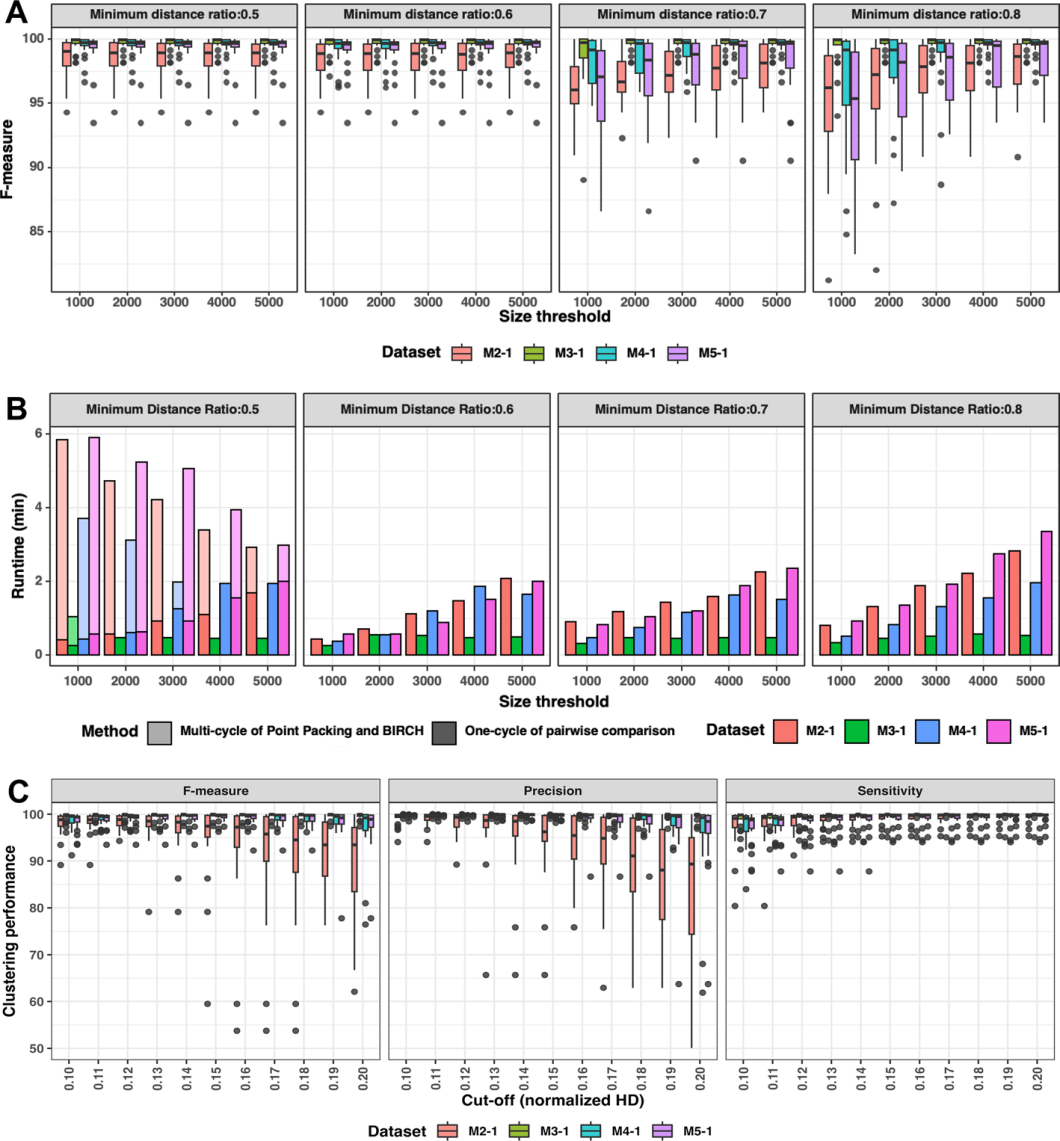
**A** Clustering performance (F-measure) of four simulated MS datasets (*M2-1, M3-1, M4-1* *and* *M5-1*) under the parameter settings of minimum distance ratios {0.5, 0.6, 0.7 and 0.8} and size thresholds {1000, 2000, 3000, 4000 and 5000}. **B** Runtime (in minutes) of four simulated MS datasets (*M2-1, M3-1, M4-1* *and* *M5-1*) under the parameter settings of four minimum distance ratios {0.5, 0.6, 0.7 and 0.8} and size thresholds {1000, 2000, 3000, 4000 and 5000}. **C** Clustering performance (F-measure, sensitivity, and precision) of four simulated MS datasets (*M2-1, M3-1, M4-1 and  M5-1*) with normalized HD cut-offs ranging from 0.1 to 0.2 with 0.01 unit increments

Data partitioning by repeated cycles of anchor generation and BIRCH clustering continues until all resulting clusters fall below a specified cluster size threshold. To determine an optimal cluster size threshold, we assessed the effect of cluster size on clustering quality. Our results suggest that the optimal choice of a clustering threshold is dependent on the minimum distance ratio during Point Packing (Fig. 3A). The use of smaller minimum distance ratio thresholds (0.5 and 0.6) resulted in above 90% F-measures for all tested cluster size thresholds {1,000, 2000, 3000, 4000 and 5,000} on four simulated MS datasets. In contrast, larger distance thresholds (0.7 and 0.8) resulted in F-measure values between 80 and 90% for lower cluster size thresholds (1,000 and 2,000).

Next, we characterized the effect of cluster size threshold and minimum distance ratio on total runtime (Fig. 3B). For a minimum distance ratio of 0.5, the total runtime was markedly longer than for higher distance ratios. This was because multiple cycles of Point Packing and BIRCH clustering were required while for minimum distance ratios above 0.5, the total runtime essentially equaled the time for pairwise comparison. The cluster size threshold was positively correlated with total runtime, which is reflective of computational cost of pairwise sequence comparison. All effects were independent of the dataset used. The fastest runtime was achieved using a minimum distance ratio of 0.6 with 1000 sequences as the size threshold in the four simulated MS datasets, and such settings were set as default in Anchor Clustering.

#### Distance threshold for hierarchical clustering

Following pairwise sequence comparison, sequences are clustered based on normalized HD using hierarchical clustering and a specified distance threshold. Relaxing the stringency of clustering by increasing the normalized HD threshold resulted in a decrease in clustering quality (Fig. 3C). This was especially obvious for the *M2-1* dataset, which showed poor F-measure and precision metrics for distance threshold values above 0.15. Raising the cut-off resulted in more sequences that were clustered erroneously, yielding a lower precision and a higher false positive rate. Conversely, lowering the cut-off separated related sequences, resulting in a lower true positive rate and lower sensitivity. The other three datasets did not demonstrate significant changes except for high cut-off values for the *M4-1* and *M5-1* datasets. For all four simulated MS datasets, a normalized HD cut-off threshold of 0.12 yielded the best performance.

#### Data fractions for model fitting

**Fig. 4:**
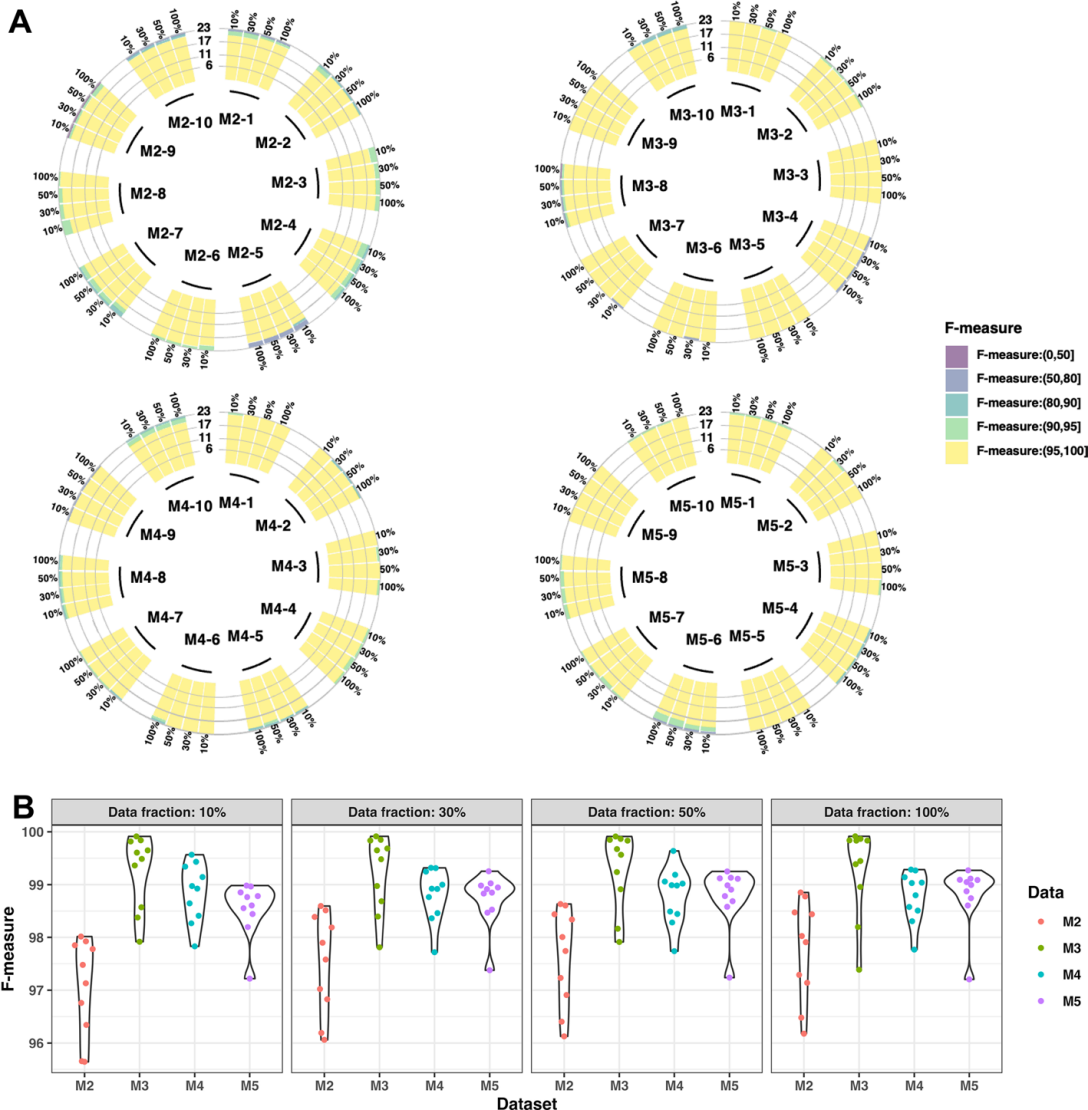
**A** The clustering performance of 40 simulated MS datasets (M2, M3, M4, and M5) was evaluated under different fractions of data {10%, 30%, 50% and 100%} that were fitted into BIRCH models. **B** The average clustering performance of each simulated MS dataset was evaluated across all junctional lengths for four fractions {10%, 30%, 50% and 100%}

To reduce memory usage, we explored the benefit of stochastically sampling a subset of sequences for constructing the BIRCH model. When comparing the clustering performance using 10%, 30%, 50% and 100% of the data to fit the BIRCH model, most junctional lengths exhibited over 95% F-measures for all four fractions of data used for model fitting (Fig. 4A). The average F-measures across all junctional lengths in 10 simulated MS datasets were consistently above 95% when utilizing different fraction settings in four groups (Fig. 4B) and a One-Way ANOVA did not yield significant differences in clustering performance among the groups. These results suggest that a fraction of 10% of the data is sufficient for fitting the BIRCH model.

### Comparison with other methods

#### Benchmarking clustering speed

**Fig. 5:**
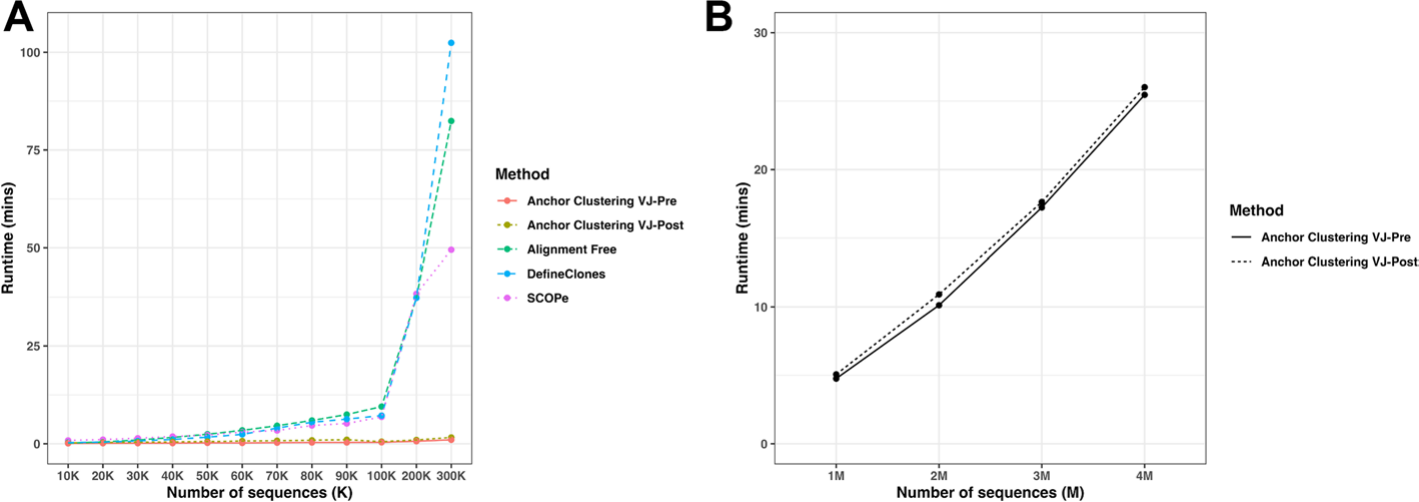
**A** Runtime comparisons (in minutes) of different BCR clustering methods on the dataset group ‘*C19’ (C19-K_Healthy_*datasets) from 10 to 100 K with 10 K increments, 200 K, and 300 K. **B** Runtime (in minutes) of Anchor Clustering on the dataset group ‘*4 Diseases Mixed’* with dataset sizes of 1 M, 2 M, 3 M and 4 M (using 10% of the data for fitting the BIRCH models) with Python version 3.10, MacBook Air Apple M2 chip with 16 GB Memory

To benchmark the runtime of Anchor Clustering, we compared its performance against three previously published BCR clustering methods (DefineClones, SCOPe and Alignment free) using non-synthetic datasets from COVID-19 and healthy individuals (Fig. 5A). All methods performed similarly up to a dataset size of 100 K sequences. However, a further increase in the dataset size resulted in an exponential, and ultimately prohibitive, increase in runtime for all methods except Anchor Clustering. To further characterize the performance of Anchor Clustering, we tested its runtime on datasets with up to 4 M sequences (Fig. 5B). By limiting the fraction of data used to fit the BIRCH models to 10%, Anchor Clustering successfully clustered datasets from 1 to 4 M sequences, and dataset ‘*4_Diseases_Mix_4M’* in less than 30 min using a MacBook Air Apple M2 chip with 16 GB Memory. Of note, one of the merged datasets (hepatitis B [27]) contained a high number of sequences with multiple V gene segment labels, which rendered the Anchor Clustering with VJ-Pre method less effective but still slightly faster than VJ-Post.

#### Benchmarking clustering quality

**Fig. 6:**
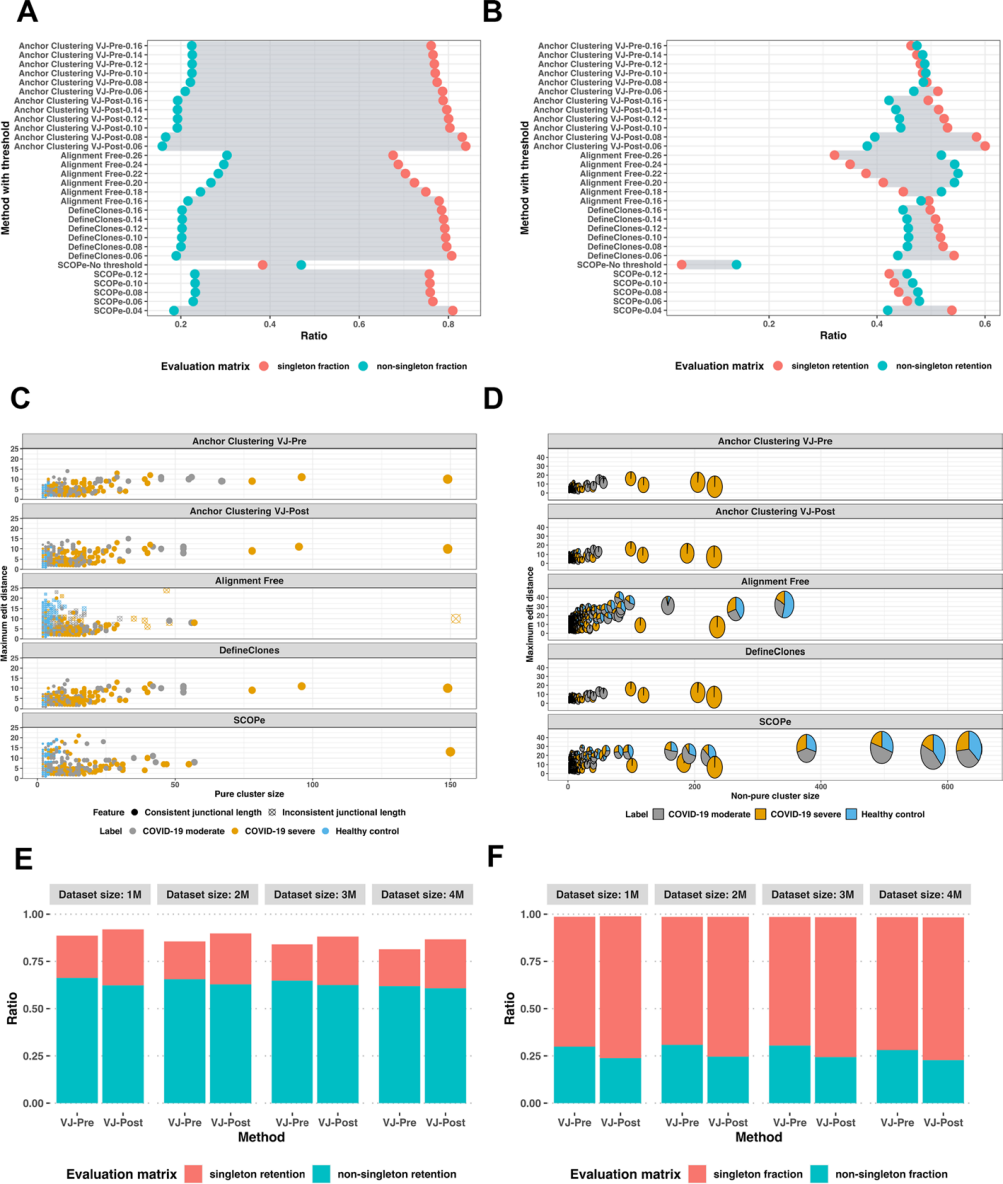
Comparisons of singleton retention and non-singleton retention ratios (**A**) and singleton fraction and non-singleton fraction ratios (**B**) with different BCR clustering methods and varying clustering thresholds on the ‘*C19-K_Healthy_100K’* dataset. Comparisons of generated pure clusters (**C**) and non-pure clusters (**D**) with benchmarking methods on the ‘*C19-K_Healthy_100K’* dataset with their optimal clustering thresholds. Comparison of singleton retention and non-singleton retention ratios (**E**) and singleton fraction and non-singleton fraction ratios (**F**) with Anchor Clustering using VJ-Pre and VJ-Post methods on the dataset group ‘4 Diseases Mixed’ with dataset size of 1 M, 2 M, 3 M and 4 M

When compared against DefineClones, Anchor Clustering exhibited a similar clustering performance on the 40 simulated datasets. For each simulated dataset, the clustering threshold for DefineClones was determined by the findThreshold function from the SHazaM R package [10]. For Anchor Clustering, the default parameters were used. The DefineClones method yielded higher F-measure and sensitivity values in 40 simulated MS datasets, while Anchor Clustering had a higher precision (Additional file 3: Fig. S3). Due to the lack of complete VDJ sequences in the 40 simulated datasets, the SCOPe and Alignment free methods could not be applied. To further benchmark clustering quality, we compared Anchor Clustering with existing BCR clustering methodologies using the ‘*C19-K_Healthy_100K’* dataset that included COVID-19 (moderate and severe) and Healthy control labels. We assessed singleton retention and non-singleton retention (Fig. 6A) and singleton fraction and non-singleton fraction (Fig. 6B) with different distance clustering thresholds for each method. Both singleton metrics reflect the clustering algorithm’s ability to retain relevant information and avoid the fragmentation of relevant data points into separate clusters. The higher the fraction and retention values, the better the performance of the clustering method. These metrics were utilized to evaluate the performance of the clustering methods in grouping similar sequences together, while also accurately separating dissimilar sequences. The Alignment free methodology with a cosine similarity threshold of 0.22 showed the highest percentage (55%) of non-singleton retention. Anchor Clustering using VJ-Pre method with a normalized HD threshold of 0.1 demonstrated the second-highest ratio (49%). SCOPe with an upper-limit distance threshold of 0.06 demonstrated the third-highest ratio (47.9%). However, the SCOPe method without a threshold was faster, but fewer sequences were correctly clustered. DefineClones with a normalized HD threshold of 0.1 and Anchor Clustering using VJ-Post with a threshold of 0.1 achieved ratios of 45.8% and 44.3%, respectively. As the clustering thresholds for each method decreased, the singleton retention ratio increased gradually. On the other hand, DefineClones and Anchor Clustering VJ-Post method showed the highest singleton fractions, accounting for approximately 80% of all the clusters compared to other methods, while the Alignment free method with varied thresholds presented the highest non-singleton fractions.

We then compared pure clusters that were generated by different methods (Fig. 6C) using their best performing settings (highest ratio of sequences to be clustered into pure clusters). Both Anchor Clustering and DefineClones showed very similar clustering results. The Alignment free approach yielded the largest pure cluster and the cluster with the largest edit distance between sequences. This is likely because the method defines clusters based on the re-weights of k-mer similarity without requiring an identical junctional length of sequences. Both, the SCOPe and Alignment free methods failed to identify the second and third largest pure ‘COVID-19 severe’ clusters, unlike the Anchor Clustering and DefineClones methods. Furthermore, Anchor Clustering with VJ-Pre was able to identify the fourth largest pure ‘COVID-19 moderate’ cluster, while Anchor Clustering with VJ-Post and the DefineClones method did not.

We also observed differences in the clustering results for non-pure clusters (Fig. 6D). The SCOPe method generated 4 large impure clusters with sizes greater than 300 sequences, which contained sequences from all three labels. Similarly, the Alignment free method also generated one large impure cluster with more than 300 sequences. Among clusters with less than 100 sequences, the Alignment free method showed higher ratios of impure clusters with three labels compared to the SCOPe method. In contrast, both Anchor Clustering and DefineClones methods demonstrated 4 intermediate sizes of ‘COVID-19 severe’ dominant clusters and fewer ratios of non-pure clusters with three different labels.

With respect to grouping sequences based on VJ usage before clustering (VJ-Pre) as opposed to the default workflow of grouping sequences after clustering (VJ-Post), we found that the clustering quality was similar for both methods across all 4 million-scale datasets (Fig. 6E and F). Anchor Clustering with VJ-Pre generally exhibited higher ratios of non-singleton retention and non-singleton fraction than VJ-Post clustering across four datasets**.** Despite these differences in performance, the ratio of impure clusters among the four datasets remained relatively consistent, with no more than 2% of all the generated clusters using both methods.

## Discussion

The widespread application of high-throughput sequencing for immune repertoire analyses is generating vast amounts of data, which represents a challenge with respect to data analysis. Clustering methods to identify clonally related sequences commonly rely on pairwise comparison of nucleotide sequences or k-mers, which is computationally intensive and potentially prohibitive for large datasets. Instead of determining the genetic distance between all sequences of a given dataset directly, Anchor Clustering partitions sequences before performing pairwise distance analyses. This is achieved by selecting a set of maximally spaced sequences as anchors and triangulating the relative position of each remaining sequence with respect to the anchor reference points. The resulting distance vectors are then clustered using unsupervised machine learning. This strategy significantly reduces the number of pairwise comparisons resulting in faster runtime and reduced memory requirements compared to existing methods.

A key consideration for determining the optimal parameter settings for Point Packing was to minimize runtime while ensuring a high quality of clustering. The minimum distance ratio, which defines the minimum distance between anchor sequences, had a significant impact on these performance metrics. A complicating factor in optimizing this parameter was that the number of generated anchors, is dependent on the size of a given dataset and the relatedness of its sequences. More closely related sequences require a lower minimum distance ratio than more distantly related sequences to obtain a given number of anchors. Increasing the minimum distance ratio between anchors resulted in fewer anchors and hence fewer sequence-to-anchor comparisons, which reduced total runtime. However, this also resulted in larger subclusters, which increased the number of partitioning cycles required to split a dataset into sufficiently small clusters for pairwise sequence comparison. In addition, for the smallest dataset *M3-1*, no anchors were obtained using a distance ratio of 0.9, suggesting that the use of high distance ratios may render the algorithm inapplicable for datasets of limited size or closely related sequences. Consequently, optimization of parameter settings for Point Packing requires the consideration of the entire workflow and reflects a balance between optimizing anchor selection by Point Packing and sequence partitioning by unsupervised clustering.

Following anchor selection, each nucleotide sequence is converted into a distance vector that reflects its genetic distance to all anchor sequences. In this study, we chose Hamming distance as a distance metric, which is straightforward to compute but requires prior partitioning of sequences based on junctional length. While the latter allowed us to improve speed by multi-processing, this approach precludes detection of similar sequences with differing length. This contrasts with methods such as the Alignment free method, which is based on k-mer frequencies and hence capable of clustering similar sequences with differing length. Using a different distance metric such as the Levenshtein distance could potentially alleviate this shortfall but was not explored in this study.

After conversion into a distance vector, the BIRCH algorithm was used to partition the dataset into subclusters. The choice of this clustering method was based its superior performance compared to other unsupervised clustering methods in initial trials. However, it is possible that other clustering methods could result in an even better performance if parameters are further optimized. An adaptation that significantly boosted performance was to only use a fraction of the data to construct the BIRCH tree model.

In addition to grouping sequences based on VJ usage after clustering, we assessed the effect of grouping sequences based on VJ usage before clustering. The potential advantage of sequence grouping pre-clustering is that the data is split into smaller subsets that are more amenable to pairwise comparison. Interestingly, this strategy yielded small but insignificant improvements in runtime with similar clustering quality.

The most significant advantage of Anchor Clustering over existing methods is that it facilitates the clustering of millions of sequences with minor hardware and software requirements. The fact that datasets with more than one million sequences using existing methods could not be clustered with the computational means used in this study may be due to the limited computational power used in this study but illustrates the potential of Anchor Clustering for large datasets. It might provide a tool to explore the immune repertoire space across a large number of individuals or species.

There are several limitations to this study. First, Anchor Clustering requires the definition of various hyperparameters that might affect performance. While we attempted to characterize a broad range of settings using synthetic and biological datasets, it is unlikely that our findings are universally applicable and individual datasets might require further refinement of hyperparameters. Second, the final step of Anchor Clustering still relies on pairwise comparison of nucleotide sequences, which could hamper the analysis of very large datasets. Third, Anchor clustering has only been tested on BCR nucleotide sequences. The use of Anchor Clustering on amino acid data will likely require modifications, such as the consideration of physicochemical features of amino acids.

## Conclusions

In summary, we present a novel clustering algorithm that can process million-scale datasets than possible with existing methods, yet still obtain similar clustering quality. Anchor Clustering could facilitate meta-analyses of immune repertoire datasets and help characterize the immune repertoire sequence space in a more comprehensive manner.

### Supplementary Information

**Additional file 1. **Dataset sources and usage details in the analyses as well as a list of the parameters used in the Anchor Clustering.

**Additional file 2. **Details about the CLA, CVO and Point Packing algorithms.

**Additional file 3. **Supplementary figures including the impact of junctional length on anchor number and runtime (Figure S1), the effect of minimum distance ratio, population size and random material rate on the number of generated anchors and runtime (Figure S2) and the comparison of clustering performance between Anchor Clustering and DefineClones using 40 simulated MS datasets (Figure S3).

## Data Availability

The source code and the data supporting of this work are available on https://github.com/skylerchang/Anchor_Clustering_Nt.

## Abbreviations

BCR: B cell receptor
BIRCH: Balanced iterative reducing and clustering using hierarchies
CDR3: Complementarity determining region 3
CLA: Conway’s lexicode algorithm
CVO: Conway’s variation operator
FN: False negative
FP: False positive
HD: Hamming distance
J: Joining
K: Kilo
M: Million
MS: Multiple sclerosis
TCR: T cell receptor
TP: True positive
V: Variable
VJ-Pre: VJ grouping pre-clustering
VJ-Post: VJ grouping post-clustering
