# Supplementary material for: Anchor Clustering for million-scale immune repertoire sequencing data
Source: BMC Bioinformatics. 2024 Jan 25;25:42. doi: 10.1186/s12859-024-05659-z (PMC10809746; doi:10.1186/s12859-024-05659-z)
Supplement: Supplementary file 2 — Additional file 2. Details about the CLA, CVO and Point Packing algorithms. [file 12859_2024_5659_MOESM2_ESM.docx]

**Algorithm 1** Conway’s Lexicode Algorithm (CLA)

# Input:

A dataset T of junctional sequences with equal length A minimum Hamming distance: *d*

# Output:

A subset S of junctional sequences from T such that every pair of sequences has Hamming distance *d* or more

# Details:

An empty set of sequences S is initialized Pass through every sequence into T in order

If the distance between the sequence and every member of S satisfies minimum Hamming distance *d*, then add this sequence into subset S

Return S

**Algorithm 2** Conway’s Variation Operator (CVO)

# Input:

Junctional sequences subsets S1, S2, with minimum Hamming distance

*d* in order

A random material rate R

A minimum Hamming distance: *d*

# Output:

Only one subset of junctional sequences with minimum Hamming distance *d*

# Details:

Two subsets S1 and S2 were combined as a union

Generate R random junctional sequences and add them to the S1 and S2 collection union as a new union

Shuffled the union into a new random order

Apply CLA on the shuffled union as a filter to retrieve a new subset of junctional sequences within the minimum Hamming distance *d*

Point Packing algorithm

Initial anchor sets were generated by CLA with randomly generated sets of junctional sequences that obey the minimum HD constraints. From these ini- tial anchor sets, new ones were created through an integration of crossover and mutation, which involves merging pairs of existing sets and introducing random junctional sequences for mutations. The population size parameter is used to profile the number of computational resources for generating initial, tentative subsets. Every subset is made up of a different number of junctional sequences that satisfy the requirement of a minimum HD. This population is kept updated multiple times in the following manner: Three subsets were chosen from the specified population size, and only two larger sizes of sets will be chosen after comparison. These two larger subsets are run through CVO to produce a new collection of sequences and this new collection replaces the smallest of the three sets chosen. If the newly created subset after CVO is smaller than the smallest subset chosen, no replacement will be made. The largest set of junctional sequences in the final population as the selected anchors are the result of Point Packing.
