# Supplementary material for: Anchor Clustering for million-scale immune repertoire sequencing data
Source: BMC Bioinformatics. 2024 Jan 25;25:42. doi: 10.1186/s12859-024-05659-z (PMC10809746; doi:10.1186/s12859-024-05659-z)
Supplement: Supplementary file 3 — Additional file 3. Supplementary figures including the impact of junctional length on anchor number and runtime (Figure S1), the effect of minimum distance ratio, population size and random material rate on the number of generated anchors and runtime (Figure S2) and the comparison of clustering performance between Anchor Clustering and DefineClones using 40 simulated MS datasets (Figure S3). [file 12859_2024_5659_MOESM3_ESM.pptx]

## Slide 1
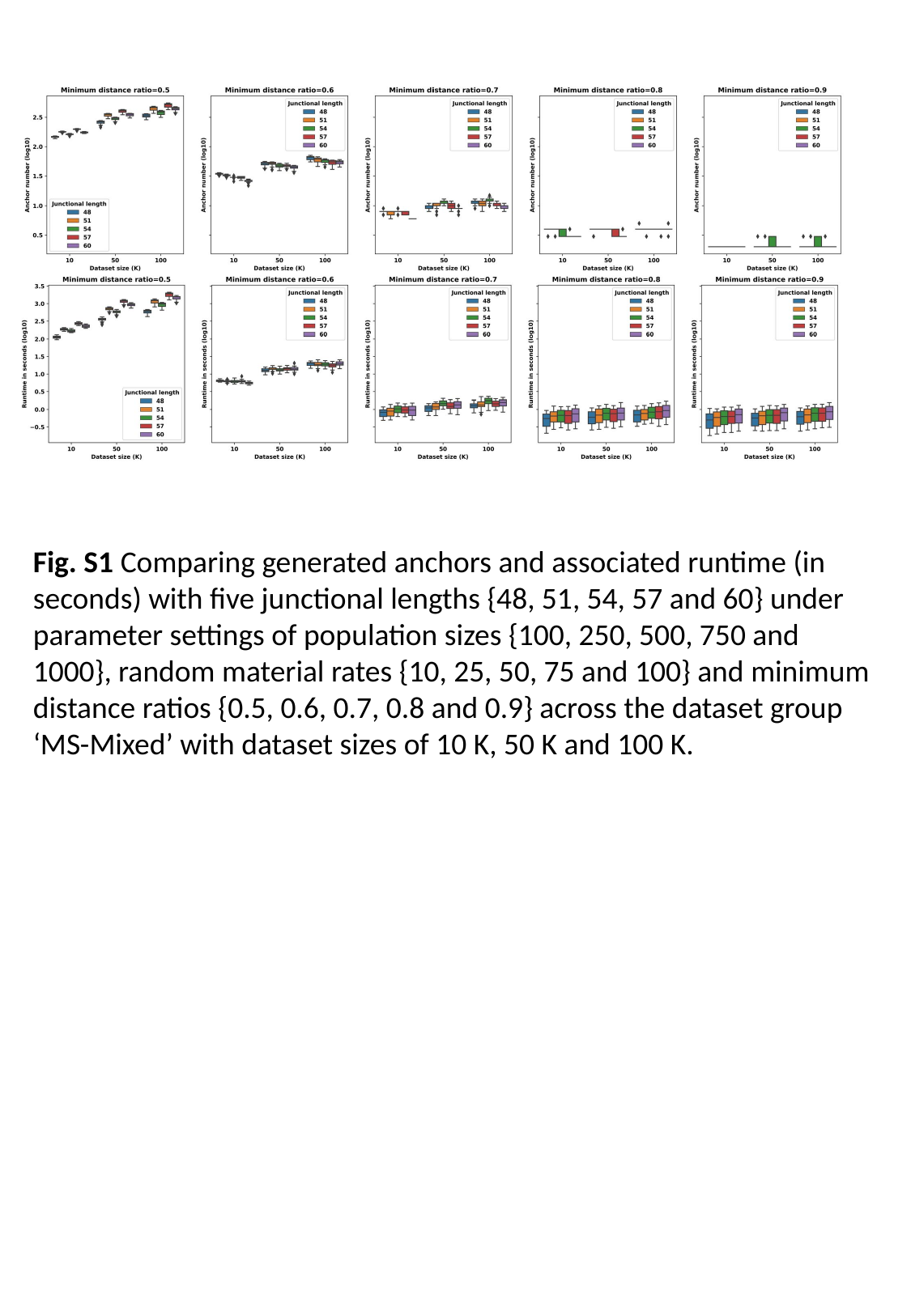

Fig. S1 Comparing generated anchors and associated runtime (in seconds) with five junctional lengths {48, 51, 54, 57 and 60} under parameter settings of population sizes {100, 250, 500, 750 and 1000}, random material rates {10, 25, 50, 75 and 100} and minimum distance ratios {0.5, 0.6, 0.7, 0.8 and 0.9} across the dataset group ‘MS-Mixed’ with dataset sizes of 10 K, 50 K and 100 K.

## Slide 2
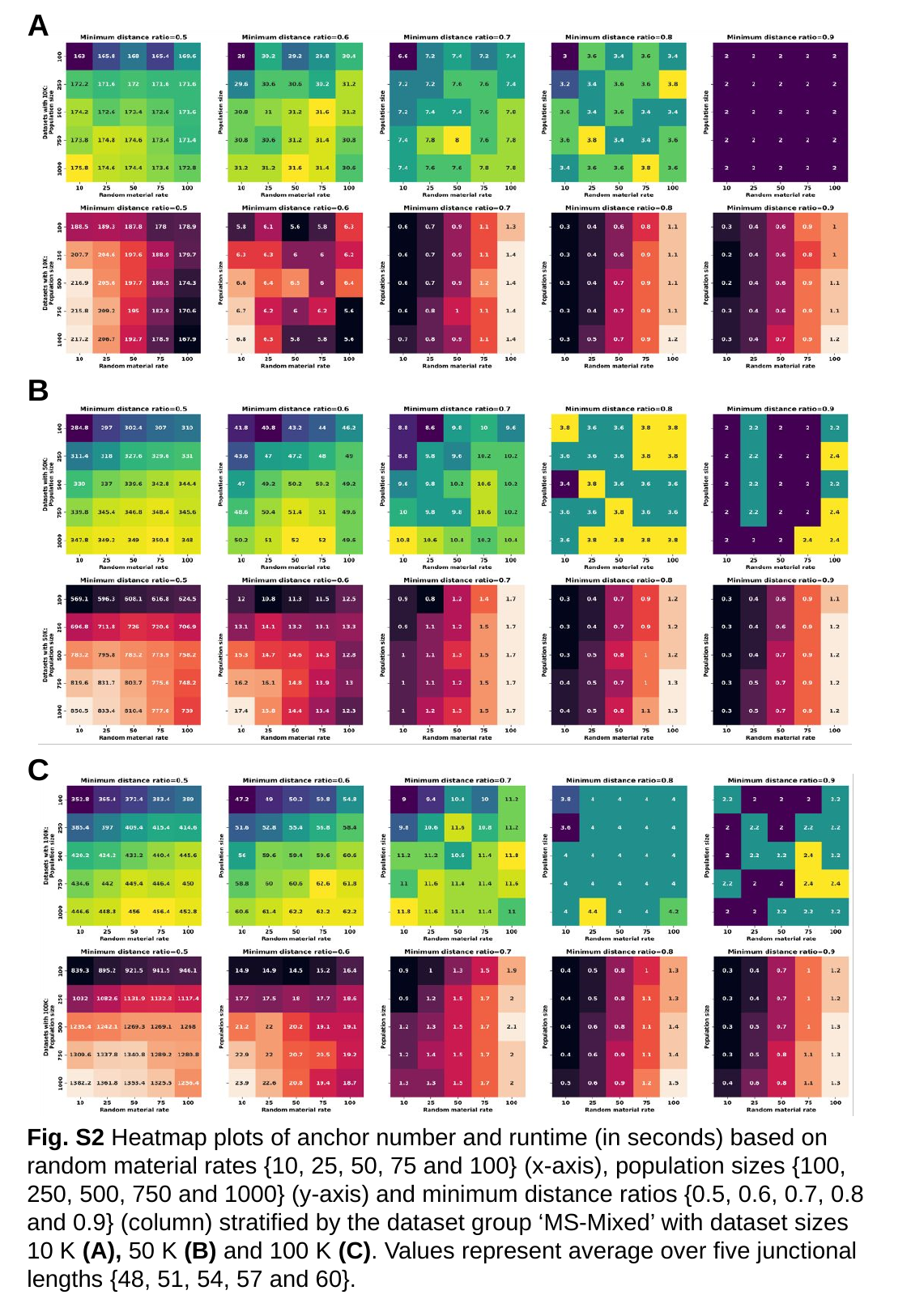

A
B
C
Fig. S2 Heatmap plots of anchor number and runtime (in seconds) based on random material rates {10, 25, 50, 75 and 100} (x-axis), population sizes {100, 250, 500, 750 and 1000} (y-axis) and minimum distance ratios {0.5, 0.6, 0.7, 0.8 and 0.9} (column) stratified by the dataset group ‘MS-Mixed’ with dataset sizes 10 K (A), 50 K (B) and 100 K (C). Values represent average over five junctional lengths {48, 51, 54, 57 and 60}.

## Slide 3
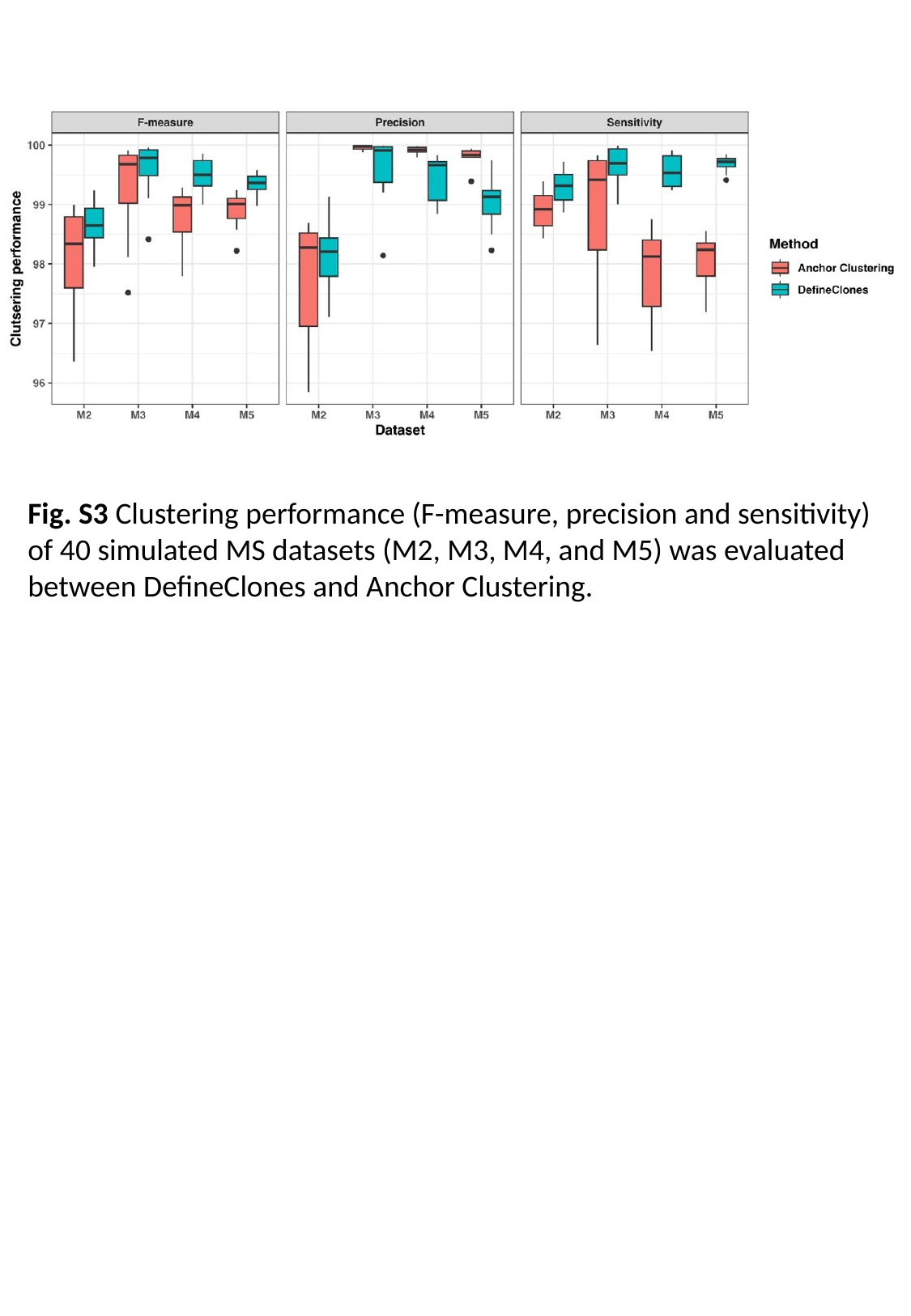

Fig. S3 Clustering performance (F-measure, precision and sensitivity) of 40 simulated MS datasets (M2, M3, M4, and M5) was evaluated between DefineClones and Anchor Clustering.
